# Targeting the Wnt/β-catenin signaling pathway in cancer

**DOI:** 10.1186/s13045-020-00990-3

**Published:** 2020-12-04

**Authors:** Ya Zhang, Xin Wang

**Affiliations:** 1 Department of Hematology, Shandong Provincial Hospital Affiliated to Shandong First Medical University, Jinan, 250021 Shandong China; 2 Department of Hematology, Shandong Provincial Hospital, Cheeloo College of Medicine, Shandong University, Jinan, 250021 Shandong China; 3 School of medicine, Shandong University, Jinan, 250021 Shandong China; 4 Shandong Provincial Engineering Research Center of Lymphoma, Jinan, 250021 Shandong China; 5 Branch of National Clinical Research Center for Hematologic Diseases, Jinan, 250021 Shandong China; 6 National Clinical Research Center for Hematologic Diseases, the First Affiliated Hospital of Soochow University, Suzhou, 250021 China

**Keywords:** Wnt/β-catenin signaling pathway, Cancer, Targeted therapy, Cancer stem cell

## Abstract

The aberrant Wnt/β-catenin signaling pathway facilitates cancer stem cell renewal, cell proliferation and differentiation, thus exerting crucial roles in tumorigenesis and therapy response. Accumulated investigations highlight the therapeutic potential of agents targeting Wnt/β-catenin signaling in cancer. Wnt ligand/ receptor interface, β-catenin destruction complex and TCF/β-catenin transcription complex are key components of the cascade and have been targeted with interventions in preclinical and clinical evaluations. This scoping review aims at outlining the latest progress on the current approaches and perspectives of Wnt/β-catenin signaling pathway targeted therapy in various cancer types. Better understanding of the updates on the inhibitors, antagonists and activators of Wnt/β-catenin pathway rationalizes innovative strategies for personalized cancer treatment. Further investigations are warranted to confirm precise and secure targeted agents and achieve optimal use with clinical benefits in malignant diseases.

## Introduction

The Wnt/β-catenin signaling pathway, also called the canonical Wnt signaling pathway, is a conserved signaling axis participating in diverse physiological processes such as proliferation, differentiation, apoptosis, migration, invasion and tissue homeostasis [1–3]. Increasing evidence indicates that dysregulation of the Wnt/β-catenin cascade contributed to the development and progression of some solid tumors and hematological malignancies [4–8].

In the Wnt/β-catenin pathway, abnormal regulation of the transcription factor β-catenin, which is the pivotal component of the Wnt signaling pathway, leads to early events in carcinogenesis [9–12]. Within the degradation complex, glycogen synthase kinase 3β (GSK3β) and casein kinase 1α (CK1α) mediate the phosphorylation of β-catenin, promoting its ubiquitination and subsequent proteasomal degradation [13, 14]. The β-catenin-dependent signaling pathway is triggered by the binding of secreted cysteine-rich glycoprotein ligands Wnts to the LRP-5/6 receptors and FZD receptors. In the presence of Wnt ligand, the binding of Wnt ligand and receptors on the cell surface induces disheveled (DVL), causing the aggregation of the complex (AXIN, GSK3β, CK1, APC) to the receptor [15]. Subsequently, the phosphorylation and inhibition of GSK3β ensure an elevation of cytosolic β-catenin concentration. Un-phosphorylated β-catenin in the cytosol migrates to the nucleus and accumulates, interacting with T cell-specific factor (TCF)/lymphoid enhancer-binding factor (LEF) and co-activators, such as Pygopus and Bcl-9, to trigger the Wnt target genes like c-Myc, cyclin D1 and CDKN1A, resulting in the upregulation of TCF/LEF target gene.

**Fig. 1:**
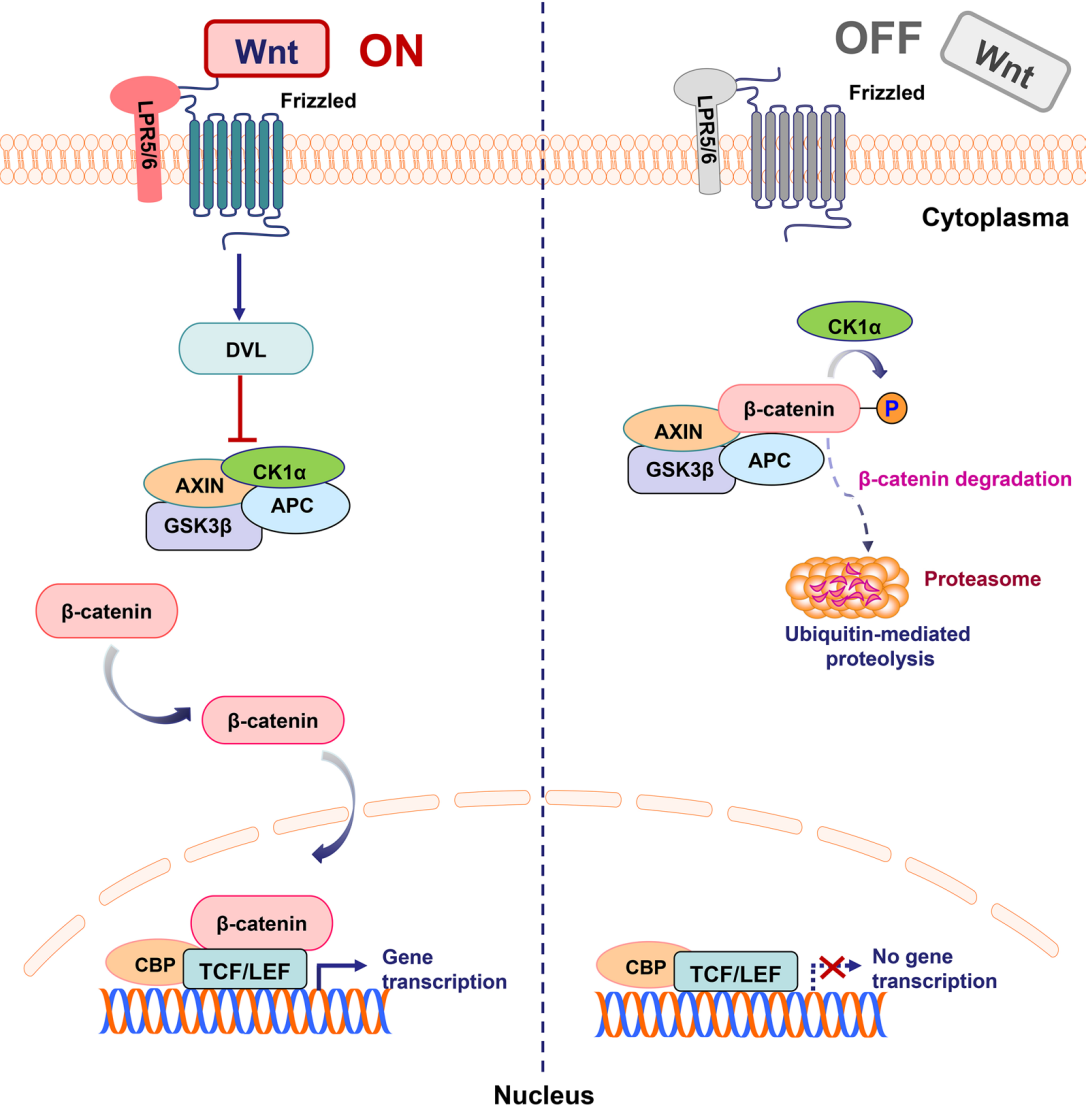
Schematic representation of activated and inhibited Wnt/β-catenin pathway. “WNT ON state”: Upon ligation of Wnts to their receptors composed of frizzled proteins and LRP5/6, the cytoplasmic protein DVL is activated and induces the suppression of GSK3β. Subsequently, stabilized β-catenin translocates into the nucleus and binds to TCF/LEF transcription factors to lead to target gene transcription. “WNT OFF state”: In the absence of WNT ligand, the destruction complex of β-catenin, a tertiary complex formed by AXIN, CK1α, GSK3β and APC, phosphorylates β-catenin, which subsequently undergoes the ubiquitin-proteasomal degradation

In addition, multiple regulatory mechanisms have been identified on the phosphorylation and ubiquitination of β-catenin by the degradation complex. Notum, which removes palmitoleate from Wnt proteins, blocks their extracellular secretion. Dickkopf (DKK) negatively regulates the initiation of Wnt protein-mediated signaling by competitively binding to LRP5/6 receptors. Besides, secreted FZD-related proteins (sFRPs), which bind to FZD receptors also blocking the initiation of Wnt protein-mediated signaling. Moreover, Wnt inhibitory factor (WIF) inhibits signaling by binding directly to Wnt proteins [16]. The transmembrane molecules ZNRF3 and RNF43 act on FZD molecules with E3 ubiquitin ligase activity [14, 17]. The 7-transmembrane receptor LGR4, LGR5 and LGR6 bind to R-spondins (RSPO) with high affinity to enhance the Wnt signal at a low dose of Wnt ligand [14, 18]. To elucidate the mechanism of Wnt/β-catenin signaling pathway activation and inhibition, a schematic diagram was depicted in Fig. 1.

Furthermore, Wnt/β-catenin signaling orchestrates multiple cell signaling cascades, such as epidermal growth factor receptor (EGFR), Hippo/YAP, nuclear factor kappa-B (NF-κB), Notch, Sonic Hedgehog and PI3K/Akt pathway, which contribute to pivotal molecular mechanism in cancer development [19–24]. EGFR could form a complex with β-catenin and promotes the invasion and metastasis of cancer cells [25, 26]. Moreover, the Hippo pathway has been shown to inhibit Dvl phosphorylation, nuclear accumulation of β-catenin and transcription of β-catenin/TCF-target genes in the Wnt/β-catenin signaling [21, 27]. Besides, the activation of Wnt/β-catenin pathway interacted with PI3K/AKT/GSK-3 cascade in glioblastoma cells and further provided mechanistic basis for the chemoresistance to temozolomide [22]. Additionally, AKT kinase could also activate β-catenin. Therefore, the cross talk between Wnt/β-catenin and PI3K-AKT pathway was confirmed to promote tumorigenesis and resistance to cancer therapy [23, 28].

Collectively, underscoring the physiological importance of Wnt/β-catenin signaling pathway in tumorigenesis, targeted agents are explored and presented promising therapeutic potential in preclinical studies and clinical trials of some cancer types. In the present review, we elaborated on the advances and challenges of Wnt/β-catenin signaling pathway targeted interventions in malignancies, aiming to provide rationales and insights on novel strategies in cancer therapy.

### Wnt/β-catenin signaling pathway interventions for cancer

**Fig. 2:**
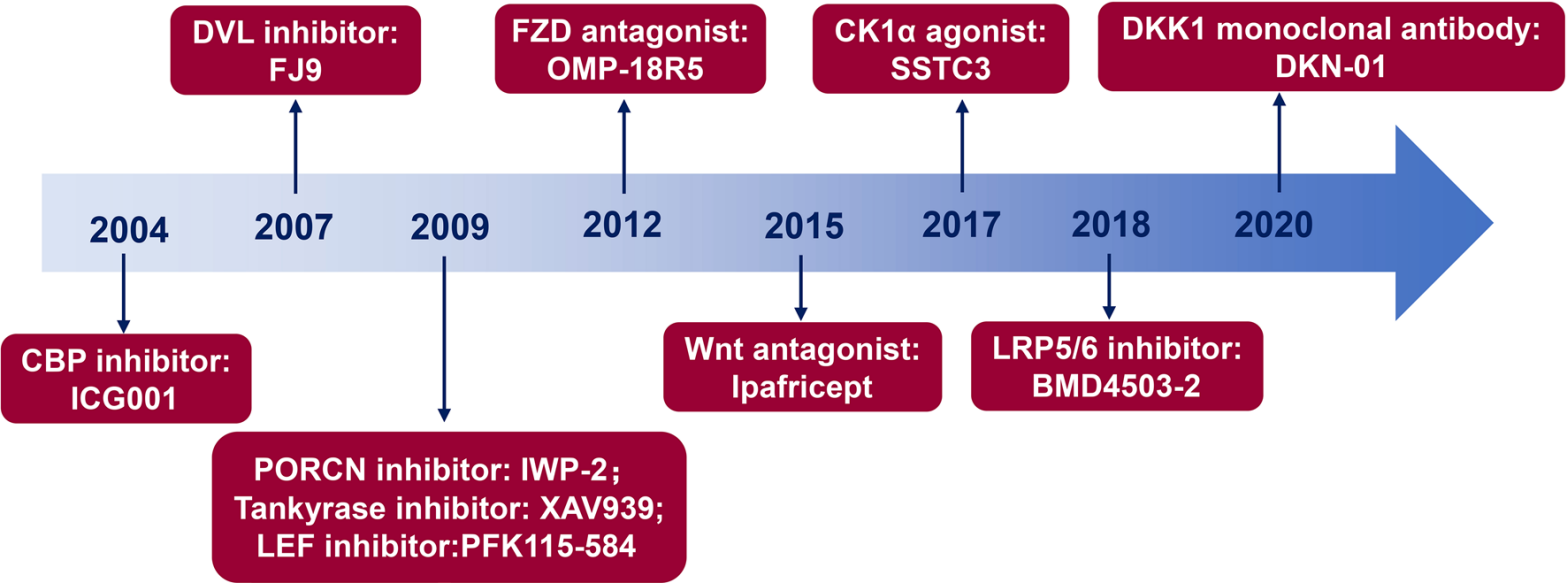
Hallmarks of diverse categories of Wnt/β-catenin targeted agents in cancer

**Fig. 3:**
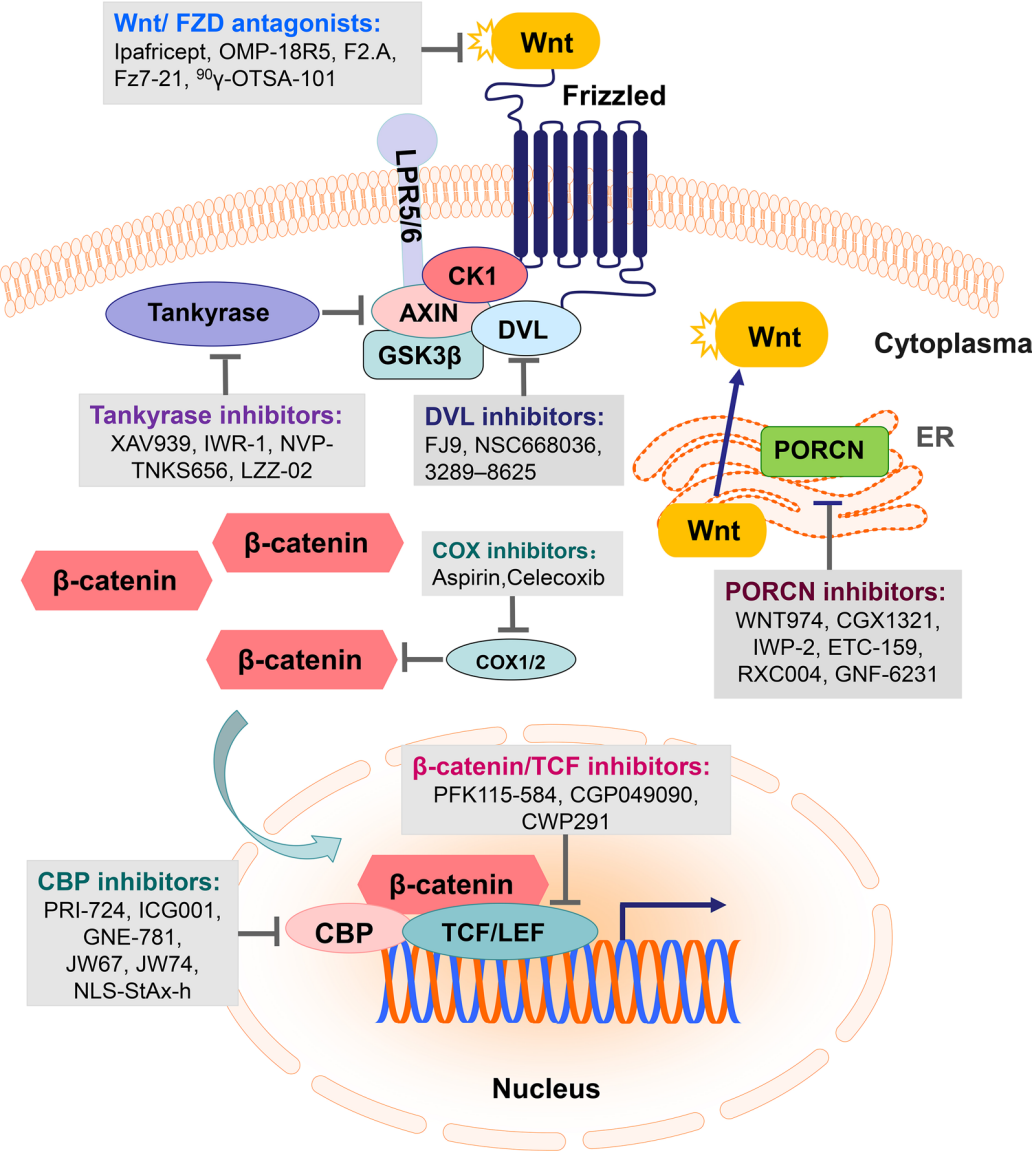
Graphic overview of Wnt/β-catenin signaling pathway targeted interventions in cancer studies. Promising therapeutics targeting Wnt ligand/ receptor interface, β-catenin destruction complex and TCF/β-catenin transcription complex are investigated in preclinical and clinical evaluations

The deregulation of Wnt/β-catenin signaling pathway is closely related to the initiation and progression of various types of cancers [4, 5, 29]. Thus, inhibitors, antagonists and agonists were designed to target this cascade in solid tumors (Table 1) and hematological malignancies (Table 2). Formulas and structures of agents targeted Wnt/β-catenin signaling pathway are listed in Additional file 1. Hallmarks of diverse categories of Wnt/β-catenin targeted agents in malignancies are illustrated in Fig. 2. In addition, Fig. 3 is plotted to present a panoramic overview of Wnt/β-catenin signaling pathway targeted interventions in cancer therapy, which was deciphered in the following aspects.

**Table 1:** Clinical trials and preclinical evaluations on Wnt/β-catenin targeted agents in solid tumors

Agents	Mechanism	Phase	Cancer type	Side effects	Identifier
WNT974	PORCN inhibitor	Phase 2	Head and neck squamous cell cancer	NR	NCT02649530
WNT974	PORCN inhibitor	Phase 1	Pancreatic cancer; colorectal cancer; melanoma; breast cancer; head and neck squamous cell cancer; cervical squamous cell cancer; esophageal squamous cell cancer; lung squamous cell cancer	NR	NCT01351103
*WNT974 (with LGX818 and Cetuximab)	PORCN inhibitor	Phase 1	Colorectal cancer	NR	NCT02278133
ETC-159	PORCN inhibitor	Phase 1	Solid tumor	Reversible hematological disorders	NCT02521844
CGX1321	PORCN inhibitor	Phase 1	Colorectal adenocarcinoma; gastric adenocarcinoma; pancreatic adenocarcinoma; bile duct carcinoma; hepatocellular carcinoma, esophageal carcinoma, Gastrointestinal cancer	NR	NCT03507998
*CGX1321 (with pembrolizumab)	PORCN inhibitor	Phase 1	Solid tumors; Gastrointestinal cancer	NR	NCT02675946
GNF-6231	PORCN inhibitor	Preclinical	Breast cancer	NR	–
^90^γ-OTSA-101	FZD10 antagonist	Phase 1	Synovial sarcoma	NR	NCT01469975
OMP-18R5	Monoclonal antibody against FZD receptors	Phase 1	Breast cancer	Nausea, alopecia, fatigue, peripheral neuropathy	NCT01973309
OMP-18R5	Monoclonal antibody against FZD receptors	Phase 1	Solid tumors	NR	NCT01345201
*OMP-18R5 (with docetaxel)	Monoclonal antibody against FZD receptors	Phase 1	Solid tumors	NR	NCT01957007
*OMP-18R5 (with nab-paclitaxel and gemcitabine)	Monoclonal antibody against FZD receptors	Phase 1	Pancreatic cancer	NR	NCT02005315
OMP-54F28	FZD8 decoy receptor	Phase 1	Solid tumors	Dysgeusia, muscle spasms, hypophosphatemia	NCT01608867
*OMP-54F28 (with sorafenib)	FZD8 decoy receptor	Phase 1	Hepatocellular cancer	Diarrhea, neutropenia and decreased appetite	NCT02069145
*OMP-54F28 (with paclitaxel and carboplatin)	FZD8 decoy receptor	Phase 1	Ovarian cancer	NR	NCT02092363
*OMP-54F28 (with nab-paclitaxel and gemcitabine)	FZD8 decoy receptor	Phase 1	Pancreatic cancer	NR	NCT02050178
Fz7-21	FZD7 antagonist	Preclinical	Gastroenteric tumor	–	–
Salinomycin	LRP5/6 inhibitor	Preclinical	Hepatocellular carcinoma; gastric cancer; colorectal cancer; bladder cancer; breast cancer	–	–
FJ9	DVL inhibitor	Preclinical	Lung cancer; melanoma	–	–
3289–8625	DVL inhibitor	Preclinical	Ovarian cancer; lung cancer	–	–
XAV939	Tankyrase inhibitor	Preclinical	Ovarian cancer; breast cancer	–	–
JW74/ JW55	Tankyrase inhibitor	Preclinical	Osteosarcoma, colon carcinoma	–	–
NVP-TNKS656	Tankyrase inhibitor	Preclinical	Hepatocellular carcinoma; colorectal cancer	–	–
LZZ-02	Tankyrase inhibitor	Preclinical	Colonic carcinoma	–	–
SSTC3	CK1α activator	Preclinical	Colorectal cancer	–	–
LF3	β-catenin/TCF	Preclinical	Colon cancer	–	–
KYA1797K/ KY1220	β-catenin	Preclinical	Colorectal cancer, breast cancer	–	–
iCRT3/5	β-catenin/TCF	Preclinical	Breast cancer; gastric cancer	–	–
ZINC02092166	β-catenin/TCF	Preclinical	Colorectal cancer	–	–
NLS-StAx-h	β-catenin/TCF	Preclinical	Colorectal cancer	–	–
*PRI-724 (with leucovorin calcium, oxaliplatin, or fluorouracil)	CBP/β-catenin antagonist	Phase 2	Colorectal cancer	Nausea, fatigue	NCT02413853
PRI-724	CBP/β-catenin antagonist	Phase 1	Pancreatic cancer	NR	NCT01764477
PRI-724	CBP/β-catenin antagonist	Phase 1	Advanced solid tumors	Nausea, vomiting, diarrhea, alopecia, fatigue, neutropenia, thrombocytopenia, neutropenic fever	NCT01302405
ICG001	CBP antagonist	Preclinical	Pancreatic cancer, lung cancer, breast cancer; ovarian cancer	–	–
Isoquercitrin	CBP antagonist	Preclinical	Colorectal cancer	–	–

**Table 2:** Clinical trials and preclinical evaluations on Wnt/β-catenin targeted agents in hematological malignancies

Agents	Mechanism	Phase	Cancer type	Side effects	Identifier
CWP291	SAM68 inhibitor	Phase 1	Relapsed or refractory AML and MDS	Nausea, vomiting, diarrhea, and infusion-related reactions	NCT01398462
PRI-724	CBP/β-catenin antagonist	Phase 2	AML; CML	NR	NCT01606579
GNE-781	CBP antagonist	Preclinical	AML	–	–
ICG001	CBP antagonist	Preclinical	AML; ALL; CML; MM	–	–
WNT974	PORCN inhibitor	Preclinical	BL		–
Wnt-C59	PORCN inhibitor	Preclinical	cHL		–
IWP-2/IWP-4	PORCN inhibitor	Preclinical	AML; cHL		–
XAV939	Tankyrase inhibitor	Preclinical	AML; T-ALL; CML		–
IWR-1	Tankyrase inhibitor	Preclinical	APL		–
Salinomycin	LRP5/6 inhibitor	Preclinical	CLL; MCL		–
iCRT14	β-catenin/TCF	Preclinical	ALL; MCL		–

### Inhibitors targeting Wnt ligand/ receptor interface

#### Porcupine inhibitors

Porcupine (PORCN), a family member of membrane-bound O-acyltransferases (MBOAT), is key for the secretion of Wnt ligands [30, 31]. Several inhibitors that target PORCN prevent the palmitoylation of Wnt proteins in the endoplasmic reticulum, which subsequently prevents their secretion [13, 24]. Blocking the acylation of WNT with a PORCN inhibitor to abolish WNT secretion becomes an effective treatment strategy. WNT974 (LGK974) is an orally available small molecule inhibitor that decreases epithelial ovarian cancer (EOC) cell viability in vitro and inhibits tumor growth in vivo [24, 32]. In EOC preclinical mouse models, WNT974 presents enhanced anti-tumor effects with the combination of paclitaxel [33]. There is currently a phase I clinical trial investigating WNT974 monotherapy for patients with pancreatic cancer, triple-negative breast cancer and cervical squamous cell carcinoma (NCT01351103). CGX1321, another PORCN inhibitor, inhibits both canonical and non-canonical Wnt signaling pathways. The single-dose escalation of CGX1321 is invested in a phase 1 clinical trial (NCT02675946) in solid tumors. In an EOC mouse model, treatment with CGX1321 led to prolonged overall survival, decreased tumor burden and increased immune cell infiltration. Furthermore, effects of some other PORCN inhibitors were evaluated in preclinical studies [34, 35]. It was reported that the combination of the PORCN inhibitor ETC-159 and the PI3K inhibitor GDC-0941 decreased RNF43-mutant pancreatic cancer cell proliferation and xenograft growth in vivo [36]. Besides, IWP-O1 was observed with significantly improved metabolic stability and inhibit the phosphorylation of DVL in Hela cells [37]. Moreover, GNF-6231 demonstrated potent inhibition activities and induced robust anti-tumor efficacy in a breast cancer mouse model [38].

#### Wnt/FZD antagonists

With the antagonism of Wnt ligands and FZD receptors, canonical Wnt signaling pathway was suppressed and indicated potential strategy in cancer therapy. Ipafricept (OMP54F28; IPA) is a recombinant fusion protein, including the cysteine-rich domain of FZD8 fused to a human IgG1 Fc fragment [39]. This structure could bind directly to Wnt ligands, competing for the binding of Wnt ligands with FZD8 receptor, thereby inhibiting Wnt regulated processes [40]. In patient-derived ovarian cancer xenograft mice models, ipafricept displayed activity to decrease the population of stem cells, suppress tumor development and promote differentiation. In addition, in preclinical studies, ipafricept exhibits synergistic anti-tumor effects combined with taxanes when given prior to chemotherapy two to three days, with 82% of the patients achieved a partial or complete response [41]. Ipafricept was also investigated in a phase 1b dose-escalation study in combination with paclitaxel and carboplatin in patients with recurrent platinum-sensitive ovarian cancer. The combination of these three agents produced similar response rates and survival outcomes compared with historical treatment regimens. Nevertheless, bone toxicities at efficacy doses prevented further testing of this treatment regimen. A phase 1b clinical trials suggested that ipafricept could also be administered with nab-paclitaxel and gemcitabine with reasonable tolerance in patients with previously untreated stage IV pancreatic cancer [42].

OMP-18R5 (vantictumab) is a monoclonal antibody targeting FZD1, FZD2, FZD5, FZD7 and FZD8 [43–45]. OMP-18R5 blocks tumor growth in xenograft mouse models of breast, pancreatic, colon, lung, and head and neck cancers and is being evaluated in a number of phase I trials for these tumor types [43, 46]. In a clinical trial, OTSA-101 was demonstrated that radioimmunotherapy targeting FZD10 is feasible in synovial sarcoma patients [47]. Besides, Pavlovic et al. utilized combinatorial antibody engineering by phage display to generate a variant antibody F2.A with specificity of FZD4 [44]. F2.A suppresses pancreatic cancer tumor growth in xenograft mouse models. Interestingly, carbamazepine, an antiepileptic drug, was recently reported to bind the cysteine-rich domain of FZD8, which suggests been explored as a promising therapy option in cancers [48]. Additionally, Fz7-21, a selective FZD7-binding peptide, disrupts intestinal stem cells and organoids, implicating the potential of therapeutic application in malignant diseases [49].

#### LRP5/6 inhibitors

As the co-receptor of Wnt, the phosphorylation of LRP5/6 promotes the activation of Wnt/β-catenin signaling pathway. The molecular complex Wnt-FZD-LRP5/6-DVL forms a structural region for AXIN interaction that disrupts degradation of β-catenin. BMD4503-2, a quinoxaline moiety, was identified as a new small-molecule inhibitor of the LRP5/6-sclerostin interaction through pharmacophore-based virtual screening and in vitro assays. The compound BMD4503-2 could revert the down-regulated activity of the Wnt/β-catenin signaling pathway through competitively binding to the LRP5/6-sclerostin complex [50].

#### DVL inhibitors

DVL is important for Wnt signal transduction by recruiting components of the β-catenin destruction complex to the cell membrane [51, 52]. DVL binds to the cytoplasmic carboxyl terminal end of FZD proteins through its PDZ domain [53]. NSC668036, FJ9, and 3289–8625 are some agents that block the DVL-PDZ interaction, resulting in subsequently inhibition of the signal transduction pathway [54, 55]. The non-electrophilic indole-2-carbinol-based chemical scaffold of FJ9 disrupted the interaction between FZD and the PDZ domain of DVL. NSC668036 and 3289–8625 were confirmed to down-regulate Wnt/β-catenin signaling and inhibit tumor cell growth in lung, colorectal and cervical cancer cell lines in vitro, as well as in a lung cancer xenografts [54].

### Agents targeting the β-catenin-destruction complex

#### Tankyrase inhibitors

Scaffolding protein AXIN is the rate-limiting component of the β-catenin destruction complex, which are constantly surveyed and regulated by tankyrases [56–58]. Tankyrases belong to the Poly (ADP-ribose) polymerases (PARPs) family, regulating the stability of AXIN1 and AXIN2 through directing AXIN ubiquitylation by RNF146 and proteasomal degradation [59, 60]. There are two isoforms, Tankyrase 1 (PARP5a) and Tankyrase 2 (PARP5b) involved in the Wnt/β-catenin signaling, increasing the degradation of AXIN by the ubiquitin–proteasome pathway [61–63]. Tankyrase inhibitor, XAV939 and IWR-1 regulated AXIN by inhibiting Tankyrase 1 and Tankyrase 2 [64, 65]. Treatment with XAV939 decreased the viability of EOC cell lines and increased radio-sensitivity in cervical cancer cells [66]. Furthermore, the tankyrase-specific inhibitor, JW74 and JW55 affects cell cycle progression and induced apoptosis and differentiation in osteosarcoma and colon carcinoma cells, respectively [67, 68]. In addition, mice xenografts and patient-derived sphere cultures of colorectal cancer (CRC) were incubated with a Tankyrase inhibitor NVP-TNKS656 combination with AKT and PI3K inhibitors. A decreased nuclear β-catenin level predicted for apoptosis suggesting the tankyrase inhibitor could overcome resistance to AKT and PI3K inhibitors [61]. The same antineoplastic effect was observed in LZZ-02, a novel Tankyrase 1/2 inhibitor [69]. Concerns of gastrointestinal toxicity have been noted in analysis of these inhibitors, and further studies are needed [70].

#### CK1 agonists

Stabilizing the β-catenin destruction complex can block the nuclear localization of β-catenin, suggesting as an attractive therapeutic target. Feasible strategy for the repositioning of existing FDA approved drugs is explored for the treatment of malignancies with deregulated Wnt signaling. For example, pyrvinium, an existing FDA approved drug, can bind all CK1 family members in vitro, selectively potentiating CK1α kinase activity [71]. Colon cancer cells with APC mutations were sensitive to pyrvinium treatment with a decrease in both Wnt signaling and cell proliferation. Pyrvinium inhibits platinum-resistant tumor growth and induces apoptosis in vitro and in vivo, and these effects are enhanced when combined with paclitaxel. Pyrvinium blocks Wnt signal by decreasing β-catenin levels and suppressing the transcription of β-catenin targeted genes. However, cancer cells with increasing level of β-catenin are no longer impacted by pyrvinium [72, 73]. In addition, a novel small-molecule CK1α activator called SSTC3 has been proved to inhibit the growth of CRC xenografts in mice and also attenuate the growth of patient-derived metastatic CRC xenograft [74, 75].

### Inhibitors targeting β-catenin/TCF transcription complex

Several compounds targeting the downstream effectors, like transcription complex and co-activators, were identified by high through-put ELISA screening, such as PFK115-584 and CGP049090, which can block the β-catenin/TCF complex in a dose-dependent manner [76]. LF3, a 4-thioureido-benzenesulfonamide derivative, robustly disrupts the critical interaction between β-catenin and the transcription factor TCF4. Besides, LF3 reduced tumor growth and induced differentiation in a mouse xenograft model of colon cancer [77]. KYA1797K/ KY1220 effectively suppressed the growth of colorectal cancer and breast cancer cells via the destabilization of both β-catenin and Ras [78–80]. Mantle cell lymphoma-initiating cells were particularly sensitive to Wnt pathway inhibitors. Targeting β-catenin-TCF4 interaction with CCT036477, iCRT3, iCRT5, iCRT14 or PKF118-310 preferentially eliminated the survival of malignant cells of acute lymphoblastic leukemia, gastric cancer, and breast cancer [81–84]. ZINC02092166 suppresses canonical Wnt signaling, downregulates the expression of Wnt target genes and inhibits the growth of colorectal cancer cells [85]. Based on the acylhydrazone component, the inhibitory activities were evaluated in cellular assays. NLS-StAx-h, a selective cell-penetrating peptide inhibitor of β-catenin-transcription factor interactions suppressed proliferation and migration of colorectal cancer cells. CWP232291 (CWP291), another small molecule inhibited Wnt-mediated transcriptional activity, was under evaluation on phase 1 clinical trial in patients with relapsed or refractory AML and myelodysplastic syndrome (MDS) [86]. Active form of CWP232204 binds to Src-associated substrate in mitosis of 68 kDa (SAM68), which regulates alternative splicing TCF, and promotes β-catenin degradation via apoptosis. Further investigations will explore CWP291, with a mechanism of aiming at eradication of earlier progenitors via Wnt pathway blockade, as combination therapy.

There are several co-activators of β-catenin-dependent transcription, including CREB binding protein (CBP). The CBPs are key transcriptional co-activators essential for a multitude of cellular processes and involved in human pathological conditions and cancer [87, 88]. Several CBP inhibitors have been developed in recent years and have shown promising antineoplastic effects in preclinical models with minimal off-target effects, such as PRI-724, ICG001, GNE-781, 1-(1H-indol-1-yl)ethenone, JW67, JW74, NLS-StAx-h, et al. [89–91]. PRI-724 is a first-in-class small molecule antagonist that inhibits the interaction between β-catenin and CBP [92]. It was phosphorylated-C-82 and was rapidly hydrolyzed to its active form C-82 in vivo [93]. In chemotherapy resistant EOC with hyperactivated CBP/β-catenin signaling, PRI-724 increased sensitization to platinum chemotherapy and preclinical studies had shown considerable toxicity profile [93, 94]. Monotherapy with ICG-001 led to the reduction of tumor-related characteristics [95, 96]. GNE-781 displayed anti-tumor activity in an acute myeloid leukemia (AML) model and was also shown to decrease Foxp3 transcript levels in a dose-dependent manner [90]. 1-(1H-indol-1-yl) ethenone markedly inhibited cell growth in several prostate cancer cell lines [89]. JW67 and JW74 were identified specifically inhibiting canonical Wnt pathway at the level of the destruction complex and inhibited the growth of colorectal cancer mouse xenograft model and multiple intestinal neoplasia mice [97]. Moreover, isoquercitrin showed anti-tumor effects on colon cancer cells (SW480, DLD-1 and HCT116), whereas exerting no significant effect on non-tumor colon cell (IEC-18), suggesting a specific effect in tumor cells in vitro [98].

### Natural agents and new activity of old drugs

It is notable that some of the natural agents exert anti-tumor activities via regulating canonical Wnt signaling pathway [99, 100]. Curcumin, isolated from the rhizome of Curcuma longa, modulates Wnt signaling pathway and exerts anti-tumor activities in melanoma, lung cancer, breast cancer, colon cancer, endothelial carcinoma, gastric carcinoma and hepatocellular carcinoma [101]. 3,3′-diindolylmethane (DIM), a natural compound derived from cruciferous vegetables inhibited proliferation of colon and colorectal cancer cells via Wnt/β-catenin pathway, highlighting as a promising chemo-preventive agent or chemo-radio-sensitizer for the prevention of tumor recurrence in cancer therapy [102]. Formononetin, isolated from the red clover, displayed anti-tumor activities in breast cancer and glioma cells with high-level IC_50_ values. To achieve high potency, formononetin was modified with a coumarin unit to design a derivate 10 via the molecular hybridization strategy. The analog 10 presented anti-proliferative effects through Wnt/β-catenin pathway in gastric cancer [103]. Besides, Wogonin, a major flavonoid compound isolated from Scutellaria radix, decreased intracellular levels of Wnt proteins and activated degradation β-catenin for proteasomal degradation [104]. Gigantol, a bibenzyl compound from orchid species, was also reported to inhibit Wnt/β-catenin signaling through down-regulation of phosphorylated LRP6 and cytosolic β-catenin in breast cancer cells [105]. Additionally, treatment of echinacoside, a phenylethanoid glycoside from Tibetan herbs, significantly reduced tumor growth and regulation of Wnt/β-catenin signaling [106]. Besides, nimbolide, a limonoid present in leaves of the neem tree, concurrently abrogated canonical Wnt signaling and induced intrinsic apoptosis in hepatocarcinoma cells [107]. Moreover, isoquercitrin, a natural flavonol compound, exerted an inhibitory effect on Wnt/β-catenin, where the flavonoid regulated downstream of β-catenin translocation to the nucleus [108]. It was also noted that triptonide, a diterpenoid epoxide presented in Tripterygium wilfordii, could effectively inhibit canonical Wnt/β-catenin signaling by targeting the downstream C-terminal transcription domain of β-catenin or a nuclear component associated with β-catenin and induced apoptosis of Wnt-dependent cancer cells [109]. Moreover, the fungus Exobasidium vexans and its subcomponent atranorin were reported to inhibit lung cancer cell motility and tumorigenesis by affecting nuclear import of β-catenin and downregulating β-catenin/LEF downstream target genes [110].

In addition, researchers had found some old drugs performed new tricks, which play important roles in tumor growth, invasion and metastasis via regulating Wnt/β-catenin signaling pathway. Carbamazepine, an antiepileptic drug, was recently reported to bind the cysteine-rich domain of FZD8, which suggested to been explored as a promising therapy option in cancers [48]. It was also reported that psychiatric agent hexachlorophene attenuated Wnt/β-catenin signaling through suppressing β-catenin degradation in colon cancer cells [111]. Salinomycin, a type of antibiotics, was reported to trigger ionic changes to inhibit proximal Wnt signaling by interfering with LPR6 phosphorylation, and thus impairing the survival of cells that depend on Wnt signaling at the plasma membrane [112–116]. Besides, hematein was found to inhibit cancer cell growth and increased apoptosis through Wnt/TCF pathway [117]. Trifluoperazine (TFP), used as an antipsychotic and antiemetics, had been found to inhibit lung CSC spheroid formation ability and suppress expression of lung CSC markers (e.g., CD44/CD133) by inhibiting Wnt/β-catenin signal transduction [118]. The similar activities were also investigated in thioridazine, pimozide and diphenylbutylpiperidine class, other antiangiogenic agents [119–121]. It is notable that cyclooxygenases (COX1 and 2) inhibitors (e.g., aspirin, celecoxib, sulindac and ursolic acid) could inhibit Wnt/β-catenin pathway in cancer cells [122–124]. Aspirin increased expression of the Wnt antagonist Dickkopf-1, which suppressed activities of cancer stem cells in CRC cells [125].

### Cancer stem cells -Wnt/β-catenin signaling pathway inhibitors

CSCs display many characteristics of embryonic or tissue stem cells and often show continuous activation of highly conserved signaling pathways related to development and tissue homeostasis [126, 127]. The Wnt/β-catenin signaling pathway is associated with regulating the pluripotency, self-renewal of stem cells and differentiation ability [1, 128].

Abnormal activation of the Wnt/β-catenin pathway promotes CSC progression and thus leads to the deterioration and metastasis of cancer [129]. For instance, abnormal activation of Wnt signaling disrupted the normal growth and differentiation of colonic crypt stem cells, resulting in a colorectal CSC phenotype by upregulating expression of target genes such as c-MYC and cyclin D [130]. Moreover, one study showed that experimental knockdown of CD146 could dedifferentiate colorectal cancer cells to acquire a stem cell phenotype through inhibiting GSK-3β which in turn promoted nuclear translocation of β-catenin for Wnt signaling activation [131]. Recent studies identified SAM68 as a novel transcriptional modulator selectively targeting CSCs over healthy stem cells via Wnt/β-catenin signaling [132]. Wnt/β-catenin signaling also exerts a crucial role in early hematopoiesis, notably in hematopoietic stem cells (HSCs). Loss- and gain-of-function studies demonstrated that Wnt signaling and β-catenin activity were necessary for proper function and cellularity control of hematopoietic cells including HSCs and MKs12-15 [133]. Overactive Wnt/β-catenin signaling led to exhaustion of HSCs, causing multilineage differentiation block and compromised hematopoietic stem cell maintenance [134].

**Fig. 4:**
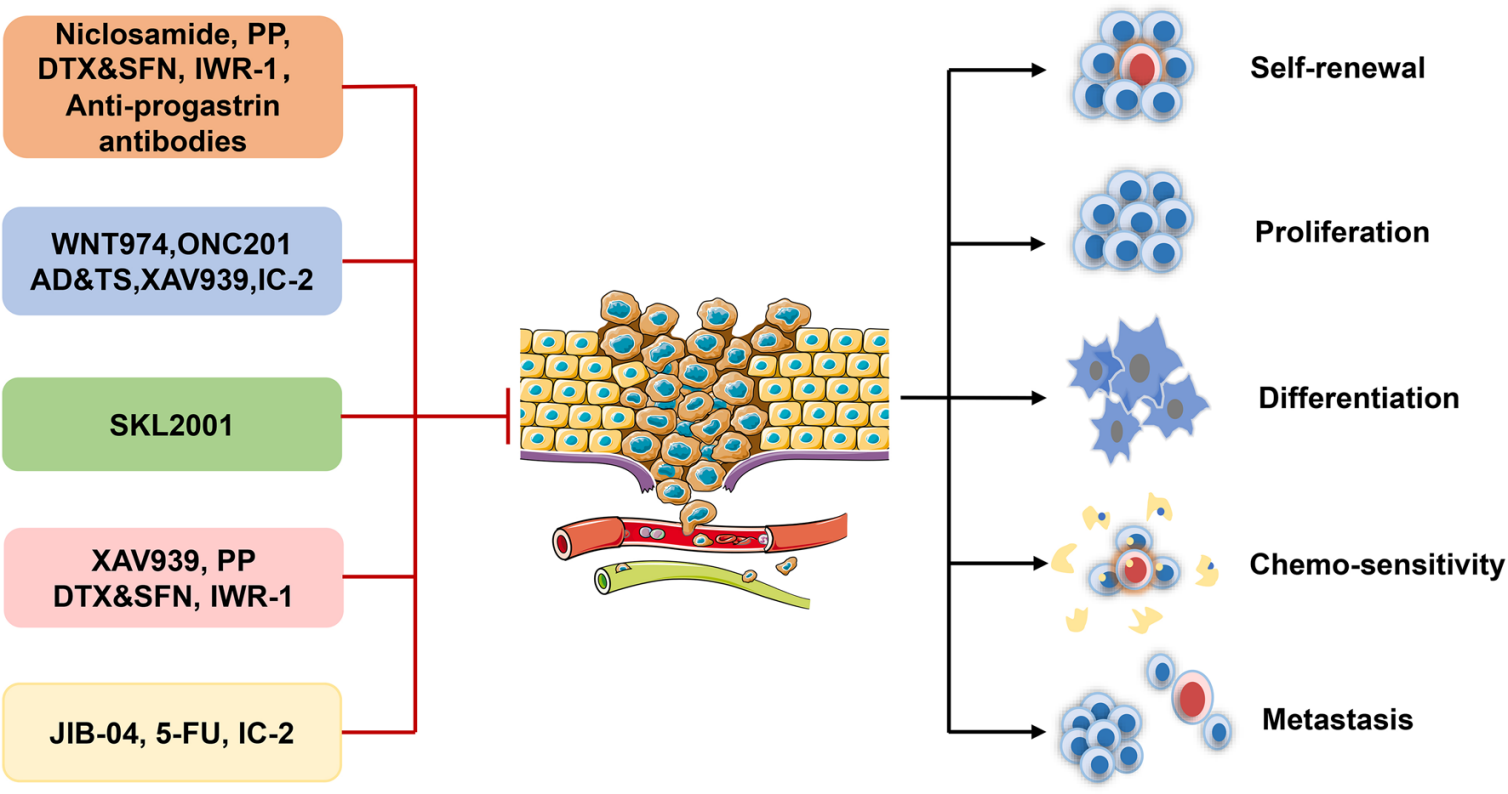
The schematic model of abnormal activation of the Wnt/β-catenin pathway promoting cancer stem cell progression and thus leading to the deterioration and metastasis of cancer

Several compounds have been identified to target CSCs via Wnt/β-catenin signaling pathway (Table 3, Fig. 4). It has been reported that PORCN inhibitor WNT974 (LGK-974) inhibited the proliferation of breast CSCs [135, 136]. Niclosamide, an FDA approved anti-helminthic agent was identified as an inhibitor of the Wnt/β-catenin pathway and showed anti-tumor properties to selectively target ovarian CSCs [137]. In addition, niclosamide decreased the level of CSCs by reducing the expression of LRP6 and β-catenin in basal-like breast cancer [138, 139]. Notably, in a phase 2 trial, the safety and effectiveness of niclosamide was proved in the treatment of colorectal cancer [140]. Furthermore, niclosamide can reduce the expression of many components in the Wnt/β-catenin signaling pathway, the self-renewal ability and population of CSCs in CRC [141]. Additionally, ONC201, which is in a phase I/II study for patients with advanced cancer (NCT02038699), induced significant CSC-suppression and repress the expression of CSC-related genes in prostate and glioblastoma tumors through suppressing the Wnt signaling pathway [142–144].

**Table 3:** Small-molecule compounds targeting Wnt/β-catenin cascade to inhibit cancer stem cells

Agents	Target	Phase	Type of cancer	Side effects	References
WNT974	PORCN inhibitor	Phase I	Breast cancer	Not reported	Solzak JP et al*.* [136]
Niclosamide	Wnt/β-catenin	Phase II	Colorectal cancer	Vomiting, diarrhea, and colitis	Burock S et al*.* [140]
	Wnt/β-catenin	Preclinical	Ovarian cancer	Not reported	Lin CK et al. [137]
	LRP6, β-catenin	Preclinical	Basal-like breast cancer	Not reported	Ye T et al. [139]
ONC201	Wnt/β-catenin	Phase I/ II	Glioblastoma cancer	Not reported	Arrillaga-Romany I et al. [144]
		Preclinical	Prostate cancer	Not reported	Lev A et al. [143]
XAV939	Tankyrase inhibitor	Preclinical	Colon cancer	Not reported	Wu X et al. [147]
		Preclinical	Head and neck squamous cell carcinoma	Not reported	Roy S et al. [146]
IWR-1	Tankyrase inhibitor	Preclinical	Osteosarcoma	Not reported	Martins-Neves SR et al. [148]
TFP	Wnt/β-catenin	Preclinical	Lung cancer	Not reported	Yeh CT et al. [118]
AD and Ts	Wnt/β-catenin	Preclinical	Lung cancer	Not reported	Lamture G et al. [165]
Chelerythrine	β-catenin	Preclinical	Non-small cell lung carcinoma	Not reported	Medvetz D et al. [150]
Wnt-C59	PORCN inhibitor	Preclinical	Nasopharyngeal carcinoma	Not reported	Cheng Y et al. [152]
IC-2	Wnt	Preclinical	Hepatocellular carcinoma	Not reported	Seto K et al
		Preclinical	Colorectal cancer	Not reported	Urushibara S et al
JIB-04	β-catenin	Preclinical	Colorectal cancer	Not reported	Kim M et al. [153]
FH535	Wnt/β-catenin	Preclinical	Pancreatic cancer	Not reported	Razak S et al. [155]
Docetaxel and sulforaphane	β-catenin	Preclinical	Breast cancer	Not reported	de Bessa Garcia SA et al. [157]
Pyrvinium pamoate	β-catenin	Preclinical	Breast cancer	Not reported	Xu L et al. [158]
SKL2001	Axin/β-catenin	Preclinical	Mesenchymal stem cell	Not reported	Jiwon Choi et al. [159]

Furthermore, many potential compounds targeting CSCs through inhibiting Wnt/β-catenin signaling pathway have been undertaken in preclinical evaluations. For example, XAV939 inhibited β-catenin signaling, thus attenuated CSC progression, thereby eliminating the CSC-mediated chemical resistance in head and neck squamous cell carcinoma (HNSCC) and colon cancer cells [145–147]. IWR-1, a tankyrase inhibitor, can hamper the expression of key stem markers in osteosarcoma, impair osteosarcoma CSC self-renewal and enhance doxorubicin sensitivity by affecting β-catenin translocation in vivo [148]. Trifluoperazine (TFP), used as an antipsychotic and antiemetics, has been found to inhibit lung CSC spheroid formation ability and suppress expression of lung CSC markers (e.g., CD44/ CD133) by inhibiting Wnt/β-catenin signal transduction [118]. Additionally, actinomycin D (AD) and telmisartan (TS) can also attenuate the number and activity of CSC and reduce CSC marker expression (such as ALDH1, SOX2 and NOS2) in lung cancer by blocking the Wnt/β-catenin signaling pathway. Besides, chelerythrine was identified to down-regulate the level of β-catenin and inhibited CSC invasion, spheroid formation and the expression of the stem marker SOX2 in non-small cell lung carcinoma (NSCLC) [149, 150]. Wnt-C59 (C59), an inhibitor of Wnt, decreased the sphere formation ability of CSCs dose-dependently in nasopharyngeal carcinoma (NPC) [151]. IC-2, a novel small-molecule Wnt inhibitor, reduced the population of CD44^+^ cells (liver CSCs) and the sphere-forming ability of hepatocellular carcinoma (HCC) cells, as well as in CRC and bladder cancer cells [152]. In addition, IC-2 increased the sensitivity of 5-FU in the DLD-1 cells, a CRC cell line. Moreover, JIB-04, a selective inhibitor of histone demethylase, significantly attenuated CSC tumor sphere formation, migration and invasion in vitro by regulating the recruitment of β-catenin [153]. A similar phenomenon was noted in FH535, which could suppress the expression of the liver CSC marker CD24 and CD44 [154, 155]. The combination of docetaxel (DTX) and sulforaphane (SFN) and pyrvinium pamoate (PP) can both inhibit the EMT (epithelial–mesenchymal transition), CSC self-renewal ability and drug resistance by decreasing β-catenin expression in BCSCs [156–158]. Additionally, SKL2001, an agonist of the Wnt/β-catenin pathway, stabilizes intracellular β-catenin via disruption of the AXIN/β-catenin interaction [159]. The treatment of mesenchymal stem cells with SKL2001 promoted osteoblastogenesis and suppressed adipocyte differentiation, providing a new strategy to regulate mesenchymal stem cell differentiation by modulation of the Wnt/β-catenin pathway. Besides, 5-FU was reported to promote stemness of colorectal cancer via p53-mediated WNT/β-catenin pathway activation [160]. Anti-progastrin humanized antibodies were investigated to decrease self-renewal of CSCs via Wnt signaling and represent potential novel strategies for K-RAS-mutated colorectal cancer [161].

### Challenges of Wnt/β-catenin signaling targeted agents in cancer

Aberrant activation of Wnt/β-catenin signaling drives oncogenic transformation in a wide range of cancers, indicating the key pathway modulators as attractive therapeutic targets in malignancies. Despite that Wnt/β-catenin targeted therapies are varied and clinical experience nascent, with the development of the targeted agents and combination strategies under investigation, the risk for off-targeting effectivity, side effects and toxicities are not allowed to be neglected. Of note, the critical role of Wnt/β-catenin signaling in stem cell maintenance raised concerns regarding the dose-limiting toxicity of targeted agents in bone, hair and gastrointestinal tract as well as in hematopoiesis, which limited of its clinical application [162–164]. Besides, considerable cross talks between the Wnt/β-catenin signaling pathway with other pathways are critical to designing effective therapeutic approaches. The combination therapy with agents that have impacts on multiple pathways in solid and hematologic malignancies needs long-term follow-up observation. Therefore, further exploration and evaluation are warranted to identify precise and safe targeted agents and achieve optimal use with clinical benefits in cancer.

## Conclusions

Novel strategies are imperative to improve the outcome of cancer patients. With great advances in the knowledge of molecular basis and the constant effort for improvement, preclinical investigations and clinical trials have been conducted on the Wnt/β-catenin signaling targeted interventions in malignancies. The Wnt/β-catenin signaling targeted regimens have been proved to represent promising candidates of individualized approaches in the treatment of cancer patients. Further investigations are expected on confirming the safety, efficacy, patient stratification and drug delivery of innovative Wnt/β-catenin targeted therapies in cancer.

## Supplementary information

**Additional file 1.** Formulas and structures of agents targeted Wnt/β-catenin signaling pathway.

## Data Availability

Not applicable.

## Abbreviations

PORCN: Porcupine
CBP: CREB binding protein
DVL: Disheveled
EOC: Epithelial ovarian cancer
CRC: Colorectal cancer
CSC: Cancer stem cells
FZD: Frizzled
LRP: Low density lipoprotein receptor-related protein
TCF/LEF: Transcription factor/ Lymphoid Enhancer Binding Factor
AXIN: Anti-Neurexin
GSK3β: Glycogen synthase kinase-3β
APC: Adenomatous polyposis coli gene
LGR: Leucine-rich repeat containing G protein-coupled receptors
RSPO: R-spondin
AML: Acute myeloid leukemia
APL: Acute promyelocytic leukemia
ALL: Acute lymphocytic leukemia
CML: Chronic myeloid leukemia
CLL: Chronic lymphocytic leukemia
MDS: Myelodysplastic Syndromes
BL: Burkitt’s lymphoma
HL: Hodgkin lymphoma
MCL: Mantle cell lymphoma
