# Supplementary material for: Targeting the Wnt/β-catenin signaling pathway in cancer
Source: J Hematol Oncol. 2020 Dec 4;13:165. doi: 10.1186/s13045-020-00990-3 (PMC7716495; doi:10.1186/s13045-020-00990-3)
Supplement: Supplementary file 1 — Additional file 1. Formulas and structures of agents targeted Wnt/β-catenin signaling pathway. [file 13045_2020_990_MOESM1_ESM.docx]

**Table S1.** **Formulas and structures of agents targeted Wnt/β-catenin signaling pathway.**

| **Agents** | **Formula** | **Structure** |
| --- | --- | --- |
| WNT974 (LGK974) | C₂₃H₂₀N₆O | 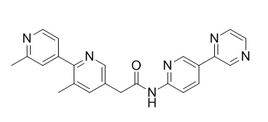 |
| ETC-1922159  (ETC159) | C_19_H_17_N_7_O_3_ | 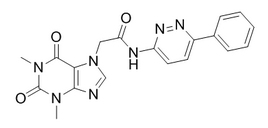 |
| CGX1321 | C_26_H_19_F_2_N_5_ | 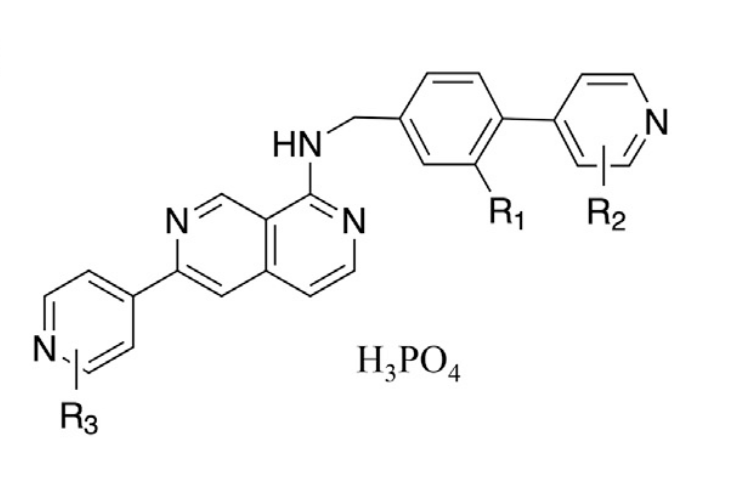 |
| PRI-724 | C_33_H_35_N_6_O_7_P | [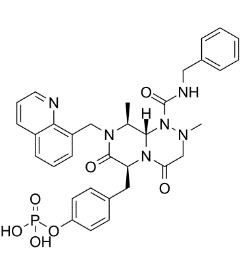](https://www.google.com/url?sa=i&url=https%3A%2F%2Fwww.medchemexpress.com%2FPRI-724.html&psig=AOvVaw3ol-ztvhkQ1mugHC4xDUGw&ust=1603626560541000&source=images&cd=vfe&ved=0CAIQjRxqFwoTCJjn7M-UzewCFQAAAAAdAAAAABAD) |
| ONC201  (TIC10) | C_24_H_26_N_4_O | 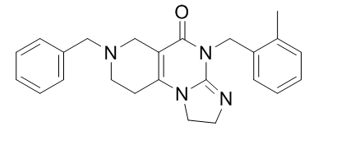 |
| Fz7-21 | C₈₅H₁₁₅N₁₈F₃O₂₅S₂ | 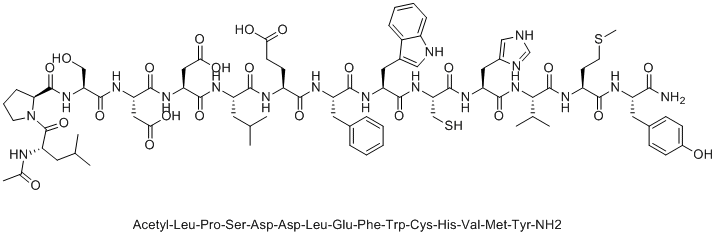 |
| Salinomycin | C_42_H_70_O_11_ | 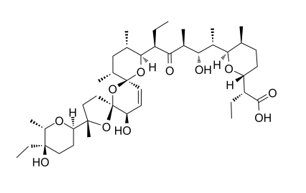 |
| 3289–8625 | C_22_N₂O_4_ | 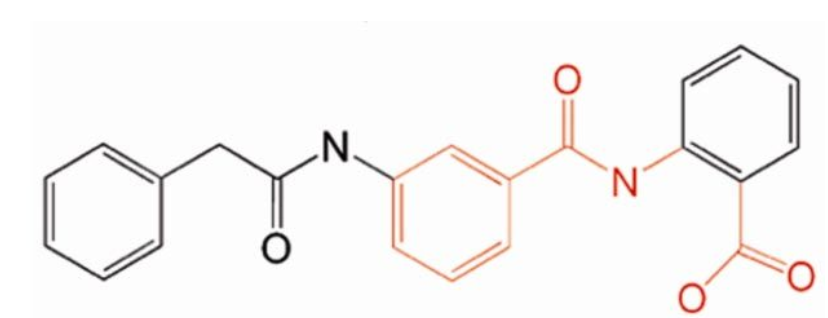 |
| XAV939 | C₁₄H₁₁F₃N₂OS | 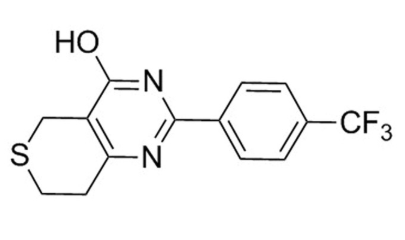 |
| JW74 | C₂₄H₂₀N₆O₂S | 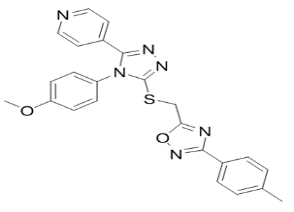 |
| JW55 | C₂₅H₂₆N₂O₅ | 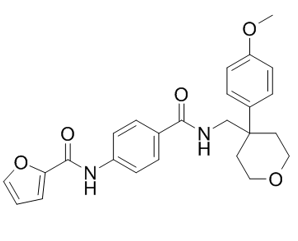 |
| NVP-TNKS656 | C₂₇H₃₄N₄O₅ | 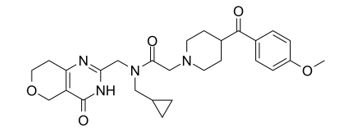 |
| LZZ-02 | C_15_HN_2_O_3_ | 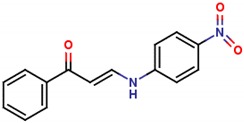 |
| SSTC3 | C₂₃H₁₇F₃N₄O₃S₂ | 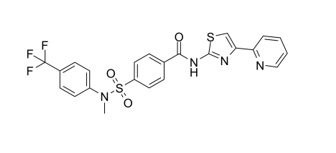 |
| LF3 | C₂₀H₂₄N₄O₂S₂ | 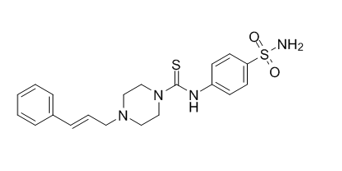 |
| KYA1797K | C₁₇H₁₁KN₂O₆S₂ | 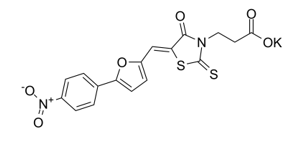 |
| KY1220 | C₁₄H₁₀N₄O₃S | 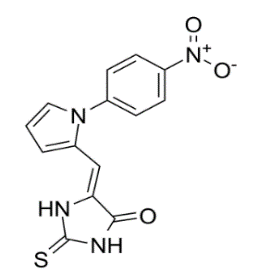 |
| iCRT3/5 | C₂₃H₂₆N₂O₂S | 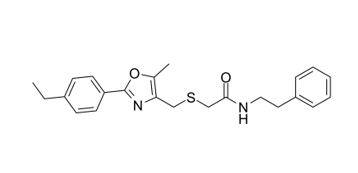 |
| ZINC02092166 | C_14_H_7_N_9_O_3_ | 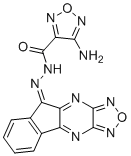 |
| NLS-StAx-h | —— | 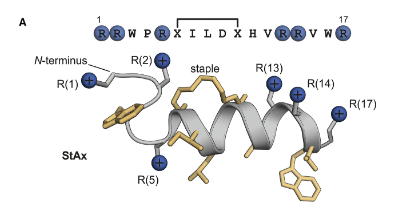 |
| ICG001 | C₃₃H₃₂N₄O₄ | 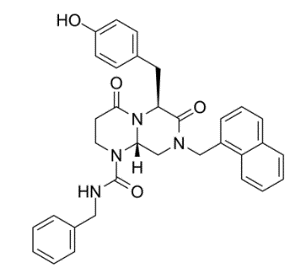 |
| Isoquercitrin | C₂₁H₂₀O₁₂ | 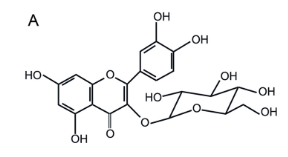 |
| GNE-781 | C₂₇H₃₃F₂N₇O₂ | 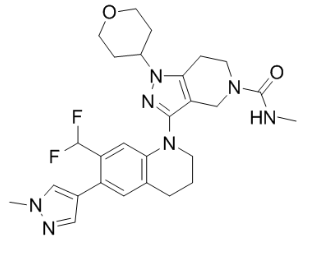 |
| Wnt-C59 | C₂₅H₂₁N₃O | 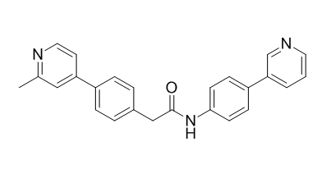 |
| IWP2 | C₂₂H₁₈N₄O₂S₃ | 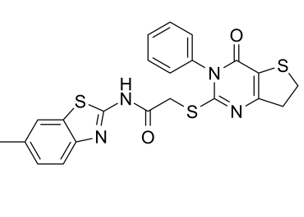 |
| IWP4 | C₂₃H₂₀N₄O₃S₃ | 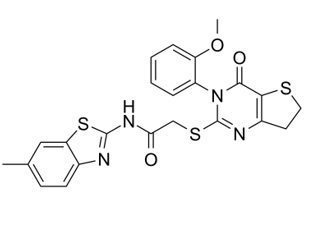 |
| IWR-1 | C₂₅H₁₉N₃O₃ | 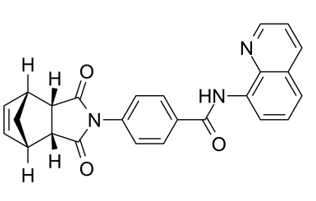 |
| iCRT14 | C₂₁H₁₇N₃O₂S | 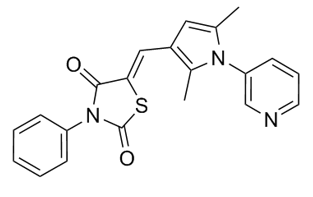 |
| Niclosamide | C₁₃H₈Cl₂N₂O₄ | 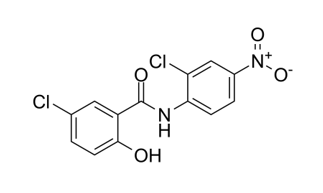 |
| Trifluoperazine | C_21_H_24_F_3_N_3_S | 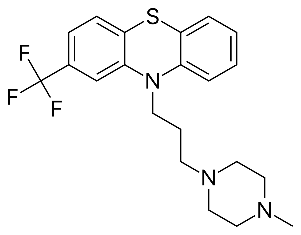 |
| Chelerythrine | C₂₁H₁₈NO₄ | 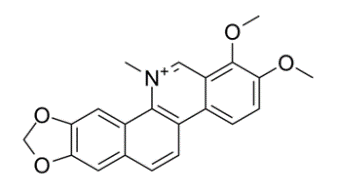 |
| IC-2 | C_33_H_32_N_4_O_3_ | 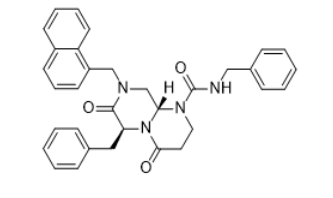 |
| JIB-04 | C₁₇H₁₃ClN₄ Cl | 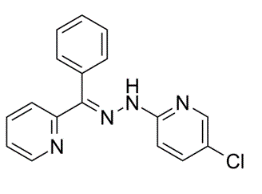 |
| FH535 | C₁₃H₁₀Cl₂N₂O₄S | **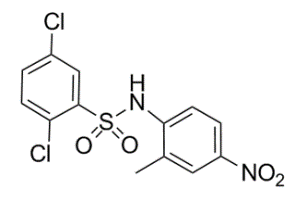** |
| Docetaxel | C₄₃H₅₃NO₁₄ | 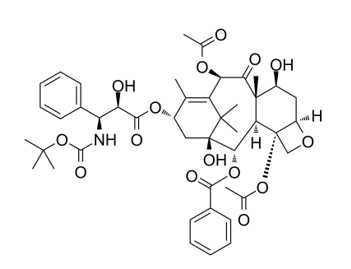 |
| Sulforaphane | C₆H₁₁NOS₂ | 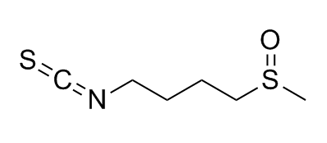 |
| Pyrvinium pamoate | C₂₆H₂₈N₃. ₁/₂ C₂₃H₁₄O₆ | 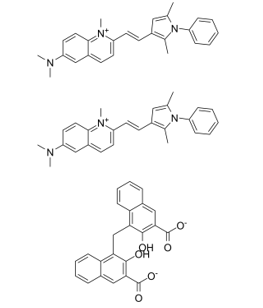 |
| SKL2001 | C₁₄H₁₄N₄O₃ | 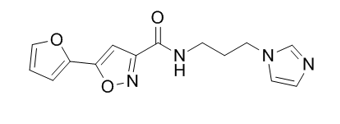 |
| Atranorin | C₁₉H₁₈O₈ | 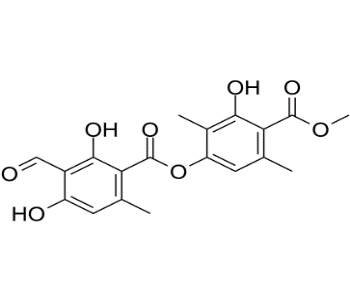 |
| Wogonin | C₁₆H₁₂O₅ | 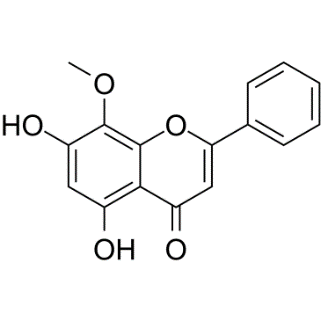 |
| Gigantol | C₁₆H₁₈O₄ | 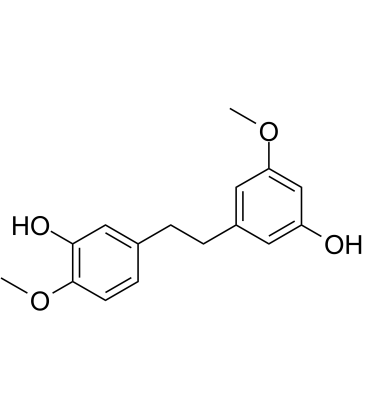 |
| Echinacoside | C₃₅H₄₆O₂₀ | 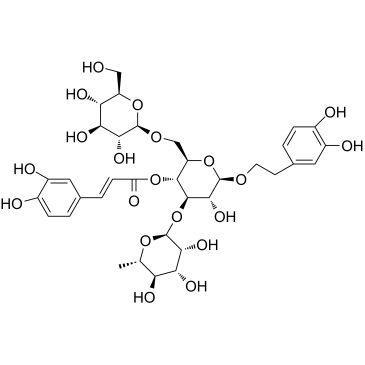 |
| Nimbolide | C₂₇H₃₀O₇ | 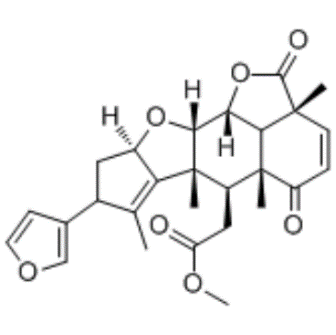 |
| Isoquercitrin | C₂₁H₂₀O₁₂ | 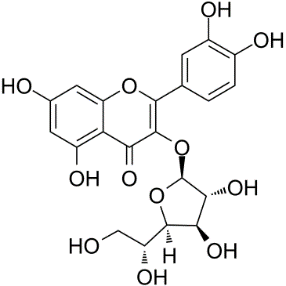 |
| Hematein | C₁₆H₁₂O₆ | 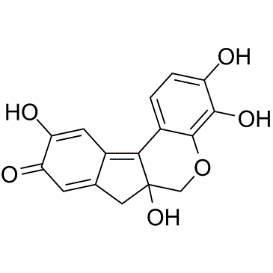 |
| Aspirin | C₉H₈O₄ | 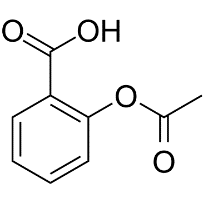 |
| Celecoxib | C₁₇H₁₄F₃N₃O₂S | 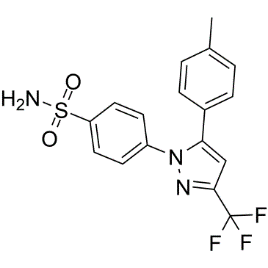 |
| Sulindac | C₂₀H₁₇FO₃S | 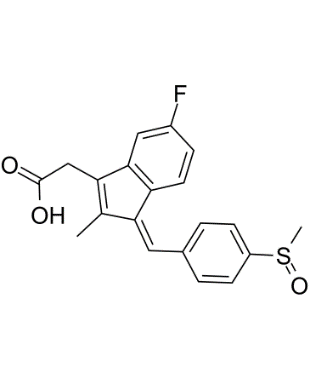 |
| Ursolic acid | C₃₀H₄₈O₃ | 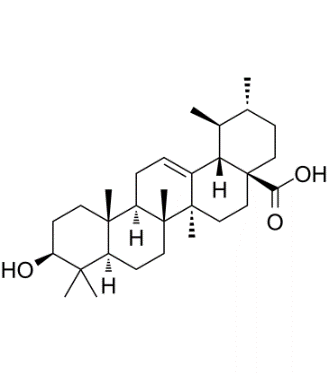 |
| Thioridazine | C₂₁H₂₇ClN₂S₂ | 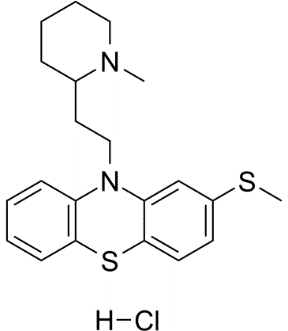 |
| Pimozide | C₂₈H₂₉F₂N₃O | 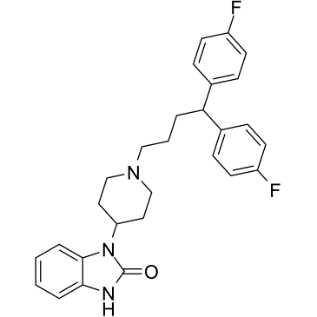 |
